# Another Step in Medical Education in Portuguese Speaking Countries

**DOI:** 10.36660/abc.20190740

**Published:** 2019-11

**Authors:** Gláucia Maria Moraes de Oliveira, Fausto J. Pinto

**Affiliations:** 1 Universidade Federal do Rio de Janeiro, Rio de Janeiro, RJ - Brazil; 2 Universidade de Lisboa, Lisboa - Portugal

**Keywords:** Cardiovascular Diseases, Education, Medical/trends, Publishing/trends, Schools,Medical, Blobal Burden of Diseases, Risk factors, Life Expectancy, Socioeconomics Factors

In 2016 the *Journal of the American Medical Association* published a theme issue on medical education, which included an editorial by Golub^1^ emphasizing the importance of looking backward on past to advance towards the future. Golub went on to say that the changes in society, such as the shifts in ethnicities, in cultural norms and in health care systems, will lead to new comprehensive, creative and innovative educational models to improve the quality of medical education and patient care.^1^

Therefore, medical training has recently undergone changes, aimed at adapting it to the new population demands, which present great challenges due to regional disparities in the health workforce distribution. Health professionals must be trained to address increasingly complex pathologies and to develop skills to lead interprofessional teams, which must be cost-effective regarding transcultural evidence, to better serve the populations taken care of.^2^

Medical training has become strategic for strengthening health systems, especially in middle- and low-income countries. The report by the Joint Learning Initiative, *Human Resources for Health: Overcoming the crisis*, and that by the World Health Organization (WHO), *World Health Report 2006: working together for health,*^3^ have shown an estimated shortage of almost 4.3 million doctors, midwives, nurses and support workers worldwide, and this shortage is even more marked in the poorest countries, especially in sub-Saharan Africa. The WHO report has proposed a ten-year action plan, in which countries can build their health workforces with the support of global partners.

Although governments in the Portuguese-speaking countries (PSC) in Africa have invested in training and retaining medical doctors in their home countries, little progress has been made.^4^ According to the 2018 WHO report, *Global strategy on human resources for health: workforce 2030*, describing a 13.1% increment in the health workforce in African countries between 2013 and 2016, there was only a small increase in the number of medical doctors in those countries.^5^That document has recommended that the WHO implement initiatives to improve the health professional education based on collaboration with countries that had developed successful models.^5^

Aiming at stimulating the development and multilateral cooperation between its Member States, the Community of Portuguese Speaking Countries, in its Strategic Plan of Cooperation for Health,^6^ has proposed the construction of cooperation networks for medical education, supporting initiatives at the undergraduate, postgraduate and research levels.^7^ That strategy reinforces the Portuguese language as the transcultural common denominator to share scientific knowledge. However, the distances, the lack of proper perspective of local problems and the financing shortage have hindered the communication between PSC, and, thus, the effective establishment of those cooperation networks.^6^

Cooperation initiatives in public health, especially those targeting communicable diseases, have faced challenges depending on factors such as the international relationships between PSC.^8-10^ In addition, it is worth considering the importance of non-communicable chronic diseases, mainly cardiovascular disease. Ischemic heart disease is the major cause of death in most PSC, with common attributable risk factors, such as diet and arterial hypertension. The genetic and cultural factors, as well as those inherent to the host, in addition to social inequalities, might explain the mortalities observed in PSC.^11,12^

The similarities between PSC could represent an opportunity for cooperation, putting into context the local demands, for the construction of exchange networks between Portuguese-speaking medical schools to enable the dissemination and adaptation of the existing models, in addition to the creation of an “Erasmus-like” Program for PSC.^13^ Therefore, a cooperation agreement between several medical schools of PSC, named Cooperation Network of Portuguese-Speaking Medical Schools (CODEM-LP) (Figure 1), will be signed this coming November in Lisbon. We are sure that the agreement will strengthen the ties of collaboration between several medical schools, contributing to the success of academic medicine in the Lusophone space.

**Figure 1:**
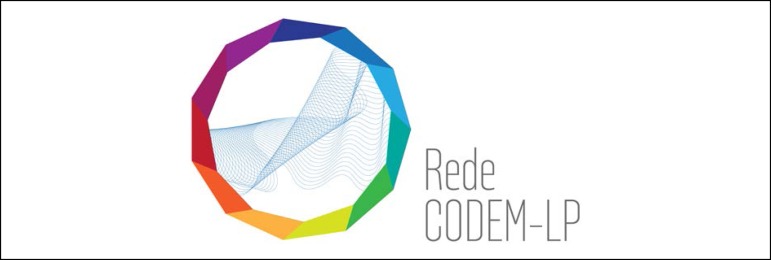
Logo of the Cooperation Network of Portuguese-Speaking Medical Schools.

## References

[1] Golub R. M. (2016). Looking Inward and Reflecting Back. JAMA,.

[2] Green M, Wayne DB, Neilson EG (2019). Medical Education 2020-Charting a Path Forward. JAMA.

[3] World Health Organization The World Health Report: working together for health.

[4] Fronteira I, Sidat M, Fresta M, The rise of medical trainingin Portuguese speaking African countries (2014). Hum Resour. Health.

[5] World Health Organization Executive Board. Human resources for health Global strategy on human resources for health: workforce 2030.

[6] Ferrinho P, Hartz Z (2016). O PECS: instrumento estruturante da reflexão e da cooperação em saúde entre os Estados membros da CPLP. An Inst Hig Med Trop.

[7] Carvalho A, IJsselmuiden C, Kaiser K, Hartz Z, Ferrinho P (2018). Towards equity in global health partnerships: adoption of the Research Fairness Initiative (RFI) by Portuguese-speaking countries. BMJ Glob Health.

[8] Carrilo ROA, Silva FRB A (2015). Fiocruz como ator da política externa brasileira no contexto da comunidade dos países de língua portuguesa: uma história revelada. Hist cienc saúde.Manguinhos.

[9] Buss PM (2018). Cooperação internacional em saúde do Brasil na era do SUS. Ciênc saúde coletiva.

[10] Fresta MJ, Ferreira MA, Delgado AP, Sambo MR, Torgal J, Sidat M, Ferrinho P (2016). Estabelecimento de uma rede estruturante da cooperação em educação médica, no âmbito do PECS-CPLP. An Inst Hig Med Trop.

[11] Nascimento BR, Brant LCC, Oliveira GMM, Malachias MVB, Reis G M A, Teixeira RA (2018). Cardiovascular disease epidemiology in Portuguese-Speaking Countries: data from the Global Burden of Disease, 1990 to 2016. Arq Bras Cardiol.

[12] Pinto FJ (2018). Cardiovascular diseases in Portuguese: the importance of preventive medicine. Arq Bras Cardiol.

[13] Oliveira GMM, Lorenzo A, Colombo FMC, Sternick EB, Brandão AA, Kaiser SE (2018). Internationalization is necessary, but is it enough?. Arq Bras Cardiol.

